# Comparison of liver biopsies before and after direct-acting antiviral therapy for hepatitis C and correlation with clinical outcome

**DOI:** 10.1038/s41598-021-93881-7

**Published:** 2021-07-15

**Authors:** Omar A. Saldarriaga, Bradley Dye, Judy Pham, Timothy G. Wanninger, Daniel Millian, Michael Kueht, Benjamin Freiberg, Netanya Utay, Heather L. Stevenson

**Affiliations:** 1 Department of Pathology, University of Texas Medical Branch, 301 University Boulevard, Galveston, TX 77555-0144 USA; 2 School of Medicine, University of Texas Medical Branch, 301 University Boulevard, Galveston, TX 77555-0144 USA; 3 Dept. of Surgery, University of Texas Medical Branch, 301 University Boulevard, Galveston, TX 77555-0144 USA; 4 Digital Pathology, Araceli Biosciences, 7425 NE Evergreen Pkwy, Hillsboro, OR 97124 USA; 5 Department of Internal Medicine, University of Texas Health Science Center at Houston, 7000 Fannin St # 1200, Houston, TX 77030 USA; 6 Department of Pathology, The University of Texas Medical Branch, 712 Texas Avenue, Clinical Services Wing-Room 5.506Q, Galveston, TX 77555-0416 USA

**Keywords:** Viral infection, Liver, Tumour virus infections

## Abstract

Direct-acting antivirals (DAA) have replaced interferon (IFN)-based therapies for hepatitis C virus. In this retrospective clinical study, we examined differences in histopathologic features in paired liver biopsies collected from the same patient before and after DAA and correlated these findings with clinical outcome. Biopsies (n = 19) were evaluated by quantitative imaging analysis to measure steatosis and fibrosis. Most patients had decreased steatosis in their post-treatment, follow-up biopsies. However, one patient had a striking increase in steatosis (from 0.86 to 6.32%) and later developed decompensated cirrhosis and hepatocellular carcinoma (HCC). This patient had a marked increase in fibrosis between biopsies, with a CPA of 6.74 to 32.02. Another patient, who already had bridging fibrosis at the time of her pre-treatment biopsy, developed cholangiocarcinoma after DAA. Even though the overall inflammatory activity in the post-treatment biopsies significantly decreased after treatment, 60% of patients had persistent portal lymphocytic inflammation. In summary, DAAs decreased steatosis and hepatic inflammation in most patients, although some may have persistence of lymphocytic portal inflammation. Patients known to have advanced fibrosis at treatment initiation and who have other risk factors for ongoing liver injury, such as steatosis, should be followed closely for the development of adverse outcomes, such as portal hypertension and primary liver cancers.

## Introduction

Viral hepatitis, among other etiologies, including non-alcoholic fatty liver disease (NAFLD), non-alcoholic steatohepatitis (NASH), and alcohol-associated liver disease (AALD), may lead to the development of chronic liver disease^1^. Globally, approximately 71 million people have chronic hepatitis C^2^, which leads to chronic inflammation, steatosis, and fibrosis. In some cases, fibrosis progresses to cirrhosis with or without the development of hepatocellular carcinoma (HCC)^3^. Enhanced fibrosis progression in hepatitis C virus (HCV)-infected patients has been associated with heavy alcohol consumption, male gender, elevated serum ALT levels, high inflammatory activity and hepatic siderosis^4,5^. Viral genotype (i.e., HCV genotype 3 and associated viral proteins), host factors (obesity, diabetes mellitus, and insulin resistance), alcohol use, and certain drug therapies (e.g., corticosteroids) may influence the development of hepatic steatosis in patients with chronic hepatitis C^6,7^.

Direct-acting antivirals (DAA), which target the viral replicative machinery, have supplanted IFN-based therapies for treating HCV. They are now the current standard therapy for the treatment of patients with HCV^8^. DAA therapy has increased treatment efficacy and has improved the safety profile when compared to IFN-based agents, in most HCV infected patients^9,10^. They have also improved treatment outcomes for patients co-infected with HCV and human immunodeficiency virus (HIV). The vast majority of patients achieve sustained virologic response (SVR) within 12 weeks post-treatment^11,12^ and liver enzymes usually return to baseline after treatment^6^. However, some patients who achieve SVR after treatment with DAAs have persistent lymphocytic portal inflammation^13^, hepatic steatosis and fibrosis^6^. Several factors, including HCV genotype, development of resistance associated variants, resistance to previous antiviral treatment (i.e., those that previously received Peg-IFN or Peg-IFN with ribavirin), HIV co-infection and the presence of cirrhosis, may decrease responses to DAA treatment^14,15^.

The effect of the DAAs on hepatic histopathological features from patients treated for HCV has not been thoroughly evaluated due to the lack of post-treatment liver biopsies. Although advanced imaging techniques are more often utilized^16^, liver biopsy remains the gold standard for grading inflammatory activity and staging the amount of fibrosis, both predictors of patient prognosis and outcome^4,17^. Early studies of DAA and HCC development were controversial. However, recent studies with larger participants have shown that even though they decrease the risk of HCC, achieving SVR does not completely eliminate a patient’s risk hepatocarcinogenesis^18^.

The goals of this retrospective study were to determine differences between liver biopsies collected from the same patient before and after DAA treatment and to correlate the histopathologic findings with clinical outcome. A strength of this study was the availability of 10 post-treatment liver biopsies to compare with each patient’s pre-treatment biopsy. Biopsies were first evaluated blindly by a board-certified pathologist (H.L.S.). Then, quantitative imaging analysis was used to measure differences in the amount of steatosis and fibrosis pre- and post-treatment. Laboratory studies pre- and post-DAA treatment were also performed. Patients were retrospectively followed to determine outcomes, including development of cirrhosis, end-stage liver disease, and cancer.

## Results

### Study patients and demographics

The goal of this study was to evaluate differences in liver biopsies collected from patients pre- and post-treatment with DAAs for HCV infection and to correlate the observations with clinical outcomes. All 10 patients achieved SVR (i.e., undetectable viral RNA for 12 weeks or more post-treatment) independent of DAA regimen (Table 1) and had paired liver biopsies (pre-and post-DAA treatment) with adequate clinical follow-up. Patient demographics, viral status, and treatment regimens are shown in Table 1. The study group consisted of both male (6/10) and female (4/10) patients with a mean age of 51.8 ± SD 7.09 years when the first biopsy was collected and a mean age of 54.7 ± SD 7.5 years when the post-treatment biopsy was collected. Sixty percent of the patients were non-Hispanic whites (6/10), 20% were Hispanic whites (2/10), and 20% were African American (2/10). Patients had a mean body mass index (BMI) of 28.2 ± SD 7.27 kg/m^2^ and 28.9 ± SD 7.49 kg/m^2^ at the time of the first and second biopsies, respectively (*P* < 0.05). The HCV viral load ranged from 6.64 × 10^5^ to 1.72 × 10^7^ IU/ml before DAA treatment. The most prevalent genotype was 1a (in 5/10 patients), which was commonly treated with the sofosbuvir/simeprevir DAA combination (in 4 of 10 patients) (Table 1).

**Table 1:** Patient demographics, viral status and treatment regimens.

HCV pt #^a^	Biopsy^b^	Age	Sex	Ethnicity/race	BMI	Genotype	Viral load (IU/mL)	DAA treatment	Treatment duration
1	Pre-Tx	58	M	Non-Hispanic/white	25.0	1a	4,641,110	Sofosbuvir/simeprevir	12 weeks
	Post-Tx	62			25.4		Not detected		
2	Pre-Tx	45	F	Non-Hispanic/white	45.0	1a	5,396,975	Sofosbuvir/ledipasvir	12 weeks
	Post-Tx	47			46.3		Not detected		
3	Pre-Tx	48	M	Hispanic/white	26.8	1a	958,240	Sofosbuvir/simeprevir	12 weeks
	Post-Tx	54			28.7		Not detected		
4	Pre-Tx	56	F	Non-Hispanic/white	32.1	2	664,007	Sofosbuvir/ribavirin	12 weeks
	Post-Tx	63			31.3		Not detected		
5	Pre-Tx	38	M	Non-Hispanic/African American	21.0	1a	2,610,582	Sofosbuvir/simeprevir	12 weeks
	Post-Tx	40			23.2		Not detected		
6	Pre-Tx	61	M	Hispanic/white	20.7	1a	3,083,562	Sofosbuvir/ledipasvir	12 weeks
	Post-Tx	62			20.6		Not detected		
7	Pre-Tx	55	F	Non-Hispanic/white	33.6	1b	3,327,711	Sofosbuvir/simeprevir	12 weeks
	Post-Tx	58			35.2		Not detected		
8	Pre-Tx	57	M	Non-Hispanic/white	23.4	2b	17,229,088	Sofosbuvir/ribavirin	12 weeks
	Post-Tx	59			25.5		Not detected		
9	Pre-Tx	53	M	Non-Hispanic/white	25.9	2b	3,964,339	Sofosbuvir/simeprevir/ribavirin	12 weeks
	Post-Tx	53			23.4		Not detected		
10	Pre-Tx	47	F	Non-Hispanic/African American	28.2	1b	2,940,158	Viekira Pak	12 weeks
	Post-Tx	49			29.0		Not detected		

HCV: hepatitis C virus; Pre-Tx: pre-DAA treatment; Post-Tx: post-DAA treatment; M: male; F: female; BMI: body mass index; IU/mL: international units/milliliter; DAA: direct-acting antiviral.

^a^All patients achieved sustained viral response.

^b^Liver biopsies were collected from patients with HCV pre- and post-DAA treatment.

### Histological changes in liver biopsies pre-and post-treatment with DAA

**Figure 1:**
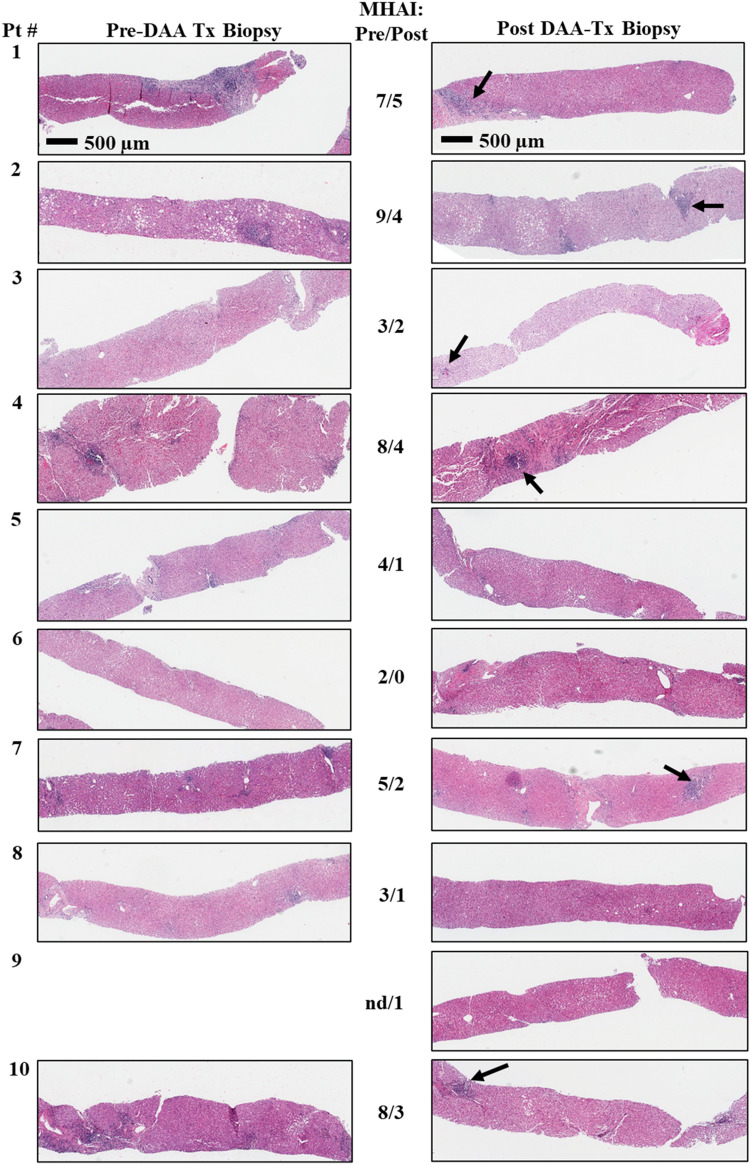
Histological comparison of liver biopsies before and after treatment with direct-acting antiviral (DAA) therapy for HCV. Percutaneous liver biopsies were collected from patients pre- and post-treatment with DAAs. Slides were stained with hematoxylin and eosin and scanned with an Aperio ImageScope Digital slide scanner. Images were collected as standard of care to determine the modified hepatitis activity index (MHAI) grading and the stage of hepatic fibrosis. The duration between the pre- and post-treatment biopsies is shown in days and ranged from 466 to 2609 days. The duration between DAA treatment and the second, post-treatment biopsy is shown in months (mos) and ranged from 6 to 28 months. Persistent lymphocytic inflammation (i.e., those with a post-treatment MHAI > 1) was observed in 60% of patients, primarily within the portal tracts (see patients 1–4, 7 and 10, →). Demographic information and treatment regimens are shown in Table 1. Differences in the MHAI scores between the pre- and post-treatment liver biopsies are shown in the center of the figure and in Table 2. MHAI: Modified hepatitis activity index; Pt: patient; Pre-Tx: pre-DAA treatment; Post-Tx: post-DAA treatment; DAA: direct-acting antiviral; nd: not done. All biopsies (1–10) are the same magnification.

**Figure 2:**
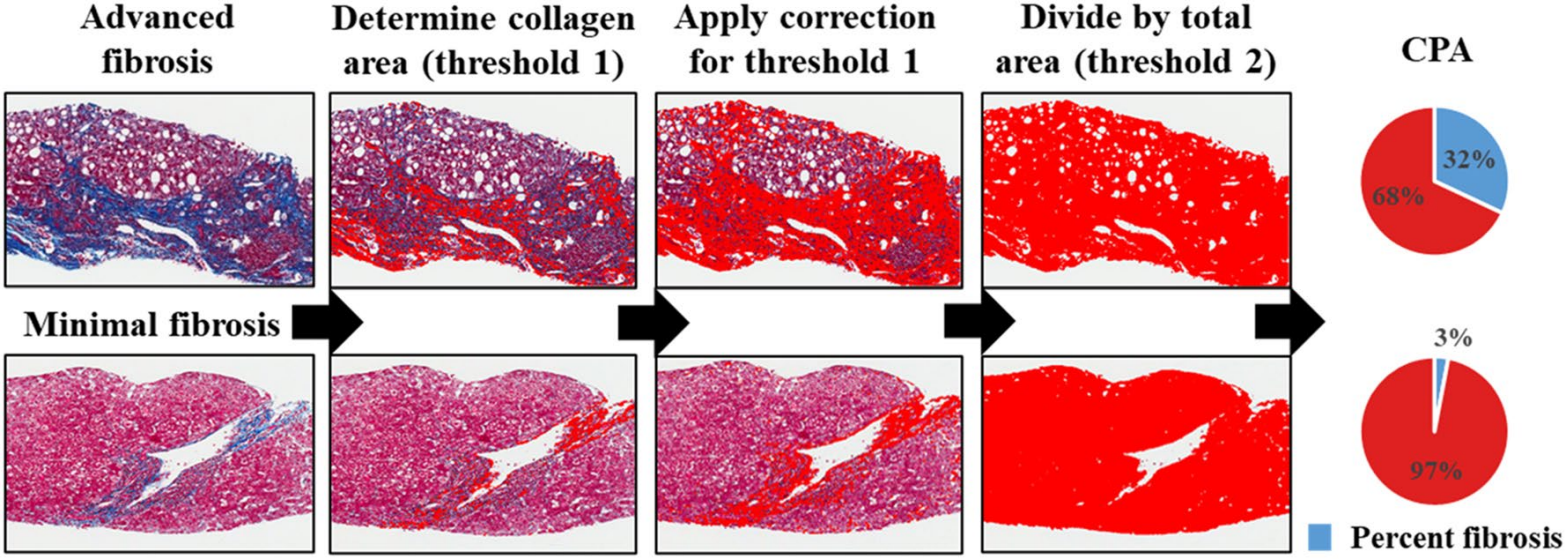
Calculation of collagen proportionate area (CPA) in patients before and after treatment. All liver biopsies from the HCV + patients were stained with Masson’s trichrome and scanned using an Aperio whole slide scanner. Low power images (×1.2) were obtained using Aperio ImageScope software. Fibrotic regions (blue areas in the trichrome stains A, E) or CPA (red areas) were determined by setting thresholds using NIS elements BR and dividing by the total surface area of the biopsy to calculate the percentage of fibrosis. The percent CPA = ∑fibrotic area/∑total area. The figure shows two representative post-treatment liver biopsies, one from a patient with advanced fibrosis (Patient 4) and one with minimal fibrosis (Patient 3). Patient 4 had a post-treatment CPA of 32.0% and patient 3 had a post-treatment CPA of 3.0%. Table 2 shows all the histopathologic findings in the patient biopsies (n = 10). CPA: Collagen proportionate area, “Eq. (1)”.

Paired liver biopsies were collected from 10 study patients pre- and post-DAA therapy and were blindly evaluated for features of inflammation, fibrosis and steatosis as described in “Materials and methods” section. All the patient’s pre- and post-treatment liver biopsies are shown in Fig. 1. Of these 10 samples, nine biopsies (patients 1–8 and 10) were collected pre-treatment and 10 were collected post-treatment. The duration between the patients’ first (i.e., pre-treatment) and second (i.e., post-treatment) liver biopsies ranged from 15.3 to 85.8 months (Table 2). The duration between DAA treatment completion and collection of their follow-up biopsies ranged from 6.0 to 28.4 months (Table 2). These were collected as standard of care to determine the modified hepatitis activity index (MHAI), the presence or absence of steatosis, and to stage the amount of hepatic fibrosis (Table 2). The fibrosis stage was determined two ways. It was first estimated using the Ishak criteria and then more objectively measured using the CPA “Eq. (1)”^19^ (Fig. 2). The Ishak fibrosis stages ranged from 1 to 6 (out of 6) prior to treatment (Table 2). The calculated CPA in the study patients prior to treatment showed percentages of fibrosis from 1.6 to 15.7% and 1.3 to 32.0%, pre- and post-treatment, respectively (Table 2). Some of the patients showed decreases in the percentages of fibrosis (patients 1, 5, 6, 8, and 10), while others showed increases (patients 2, 3, 4, and 7), post-DAA treatment (Table 2). Persistent lymphocytic inflammation (i.e., those with a post-treatment MHAI > 1) was primarily observed within the portal tracts, in 60% of the patients (patients 1–4, 7, 10) (Table 2 and Fig. 1).

**Table 2:** Histopathological findings in liver biopsies pre- and post-DAA therapy.

HCV pt #	Biopsy^a^	MHAI^b^ (n/18)	Fibrosis^c^ stage (n/6)	CPA^c^ %	Steatosis %^d^ (pathologist)	Steatosis %^d^ (visiopharm)	Duration between pre- and post-Tx Bx (mos)	Duration between SVR and post-Tx Bx (mos)
1	Pre-Tx	7	3	14.911	1	0	54.8	14.2
	Post-Tx	5	2.5	13.461	1	0		
2	Pre-Tx	9	2	5.299	40	6.994	16.6	9.4
	Post-Tx	4	2	11.596	20	1.555		
3	Pre-Tx	3	1	1.613	0	0	70.2	17.5
	Post-Tx	2	0.5	3.009	0	0		
4	Pre-Tx	8	6	6.744	5	0.861	85.8	28.4
	Post-Tx	4	6	32.024	60	6.321		
5	Pre-Tx	4	1	6.661	0	0	20.8	19.2
	Post-Tx	1	1	3.496	0	0		
6	Pre-Tx	2	1	3.677	0	0	15.3	6.0
	Post-Tx	0	1	2.905	0	0		
7	Pre-Tx	5	3	5.781	10	1.231	26.8	24.1
	Post-Tx	2	1	6.767	10	2.735		
8	Pre-Tx	3	2	8.021	15	0.183	24.6	20.1
	Post-Tx	1	1	1.265	< 5	0.597		
9	Pre-Tx	nd	nd	nd	nd	nd	nd	26.0
	Post-Tx	1	1.5	7.461	10	nd		
10	Pre-Tx	8	4.5	15.664	5	1.248	21.1	15.3
	Post-Tx	3	4	14.362	2	0.201		
*P value		0.001	0.063	1.0	0.875	0.459		

DAA: direct-acting antiviral; HCV: hepatitis C virus; Pt: patient; Pre-Tx: pre-DAA treatment; Post-Tx: post-DAA treatment; MHAI: modified hepatitis activity index; CPA: collagen proportionate area; Bx: biopsy; Mos: months; nd: not done.

*P values < 0.05 were considered statistically significant.

^a^Liver biopsies were collected from patients with HCV pre- and post-DAA treatment.

^b^We used the MHAI to grade the amount of hepatitis activity (scale of 0 to 18).

^c^Fibrosis staging was first evaluated using light microscopy and the Ishak criteria (scale of 0 to 6). Then, CPA was calculated for each liver biopsy using a previously published method^19,25,26^. The percentage of hepatocytes involved by macrovesicular steatosis was first estimated by a pathologist (H.L.S.) and then quantified using a custom trained algorithm “Eq. (2)” (Visiopharm).

**Figure 3:**
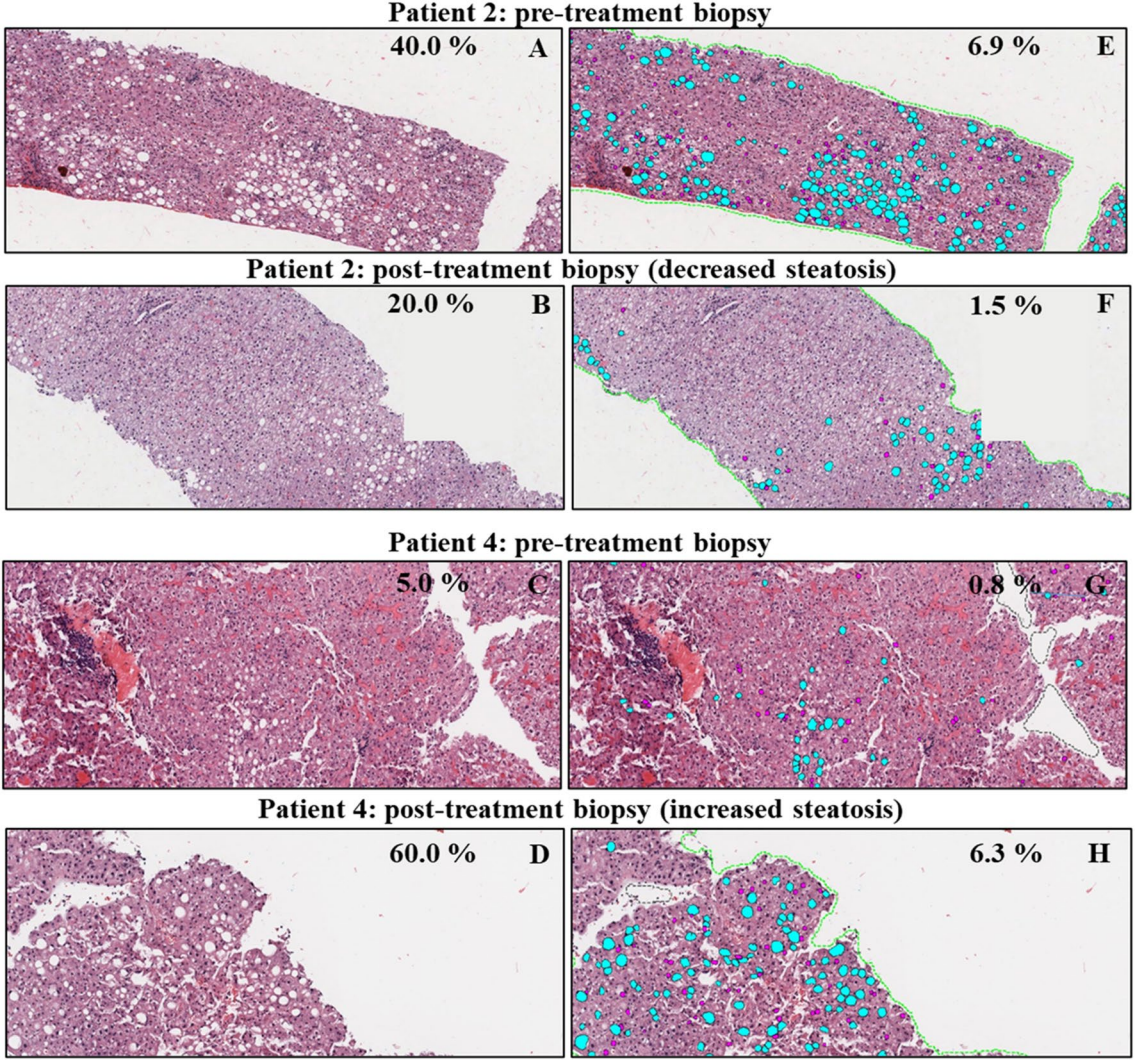
Quantitative imaging analysis highlights differences in hepatic steatosis before and after DAA treatment for viral hepatitis C. H&E-stained slides were digitally scanned on an Aperio ImageScope Digital slide scanner at low-power (×1.2). We used an algorithm developed by Visiopharm (imaging analysis software) to more objectively quantity steatosis droplets in the liver biopsies. Lipid droplets that were considered as macrovesicular steatosis droplets measured > 250 square µm in greatest dimension (turquoise-colored circles) and microvesicular steatosis “Eq. (2)” droplets were those that measured < 250 square µm (magenta circles). All lipid droplets smaller than 25 square microns were removed to diminish background. Vessels and artifacts in the biopsy tissue were marked with black dashed lines (see **G** and **H**), allowing them to be removed from analysis. The entire core was outlined by the software, which enabled measurement of the surface area of each biopsy. Patients 2 and 4 are shown as representative examples of patients whose post-treatment liver biopsies showed decreased and increased macrovesicular steatosis when compared to their pre-treatment biopsies, respectively. Patient 2 (**A**,**B**) presented with more macrovesicular steatosis pre-treatment (6.99% using Visiopharm analysis **E**) than post-treatment (1.56% using Visiopharm analysis **F**). Patient 4 (**C**,**D**) had minimal macrovesicular steatosis pre-treatment (0.86% using Visiopharm analysis **G**) that increased substantially post-treatment (6.32% using Visiopharm analysis H). Table 2 shows all the histopathologic findings in the patient biopsies (n = 10). Magnification: (**A**) and (**E**), ×40; (**B**) and (**F**), ×100; (**C**,**D**) and (**G**,**H**) ×400. Pre-: pre-DAA treatment; Post: post-DAA treatment.

Since hepatic steatosis may be caused by HCV infection itself^20^ or may be caused by other risk factors, such as obesity and metabolic syndrome^21^, we compared the amount of steatosis present in each patient’s liver biopsy before and after DAA treatment using traditional microscopy (Fig. 3A–D). Five study patients had macrovesicular steatosis that was estimated to involve greater than or equal to 5.0% of hepatocytes prior to DAA treatment (Table 2 and Figs. 1 and 3) (patients 2, 4, 7–8, and 10). The estimated percentage of hepatocytes involved by macrovesicular steatosis decreased in three patients (patients 2, 8, and 10), remained stable in one patient (patient 7), and increased in one patient (patient 4), post-treatment (Table 2). The percentage of hepatic steatosis “Eq. (2)” was more objectively calculated in these five study patients using a quantitative imaging software algorithm (Visiopharm), as described in the methods, “Quantification of hepatic steatosis” section (Fig. 3E–H) and other studies^22–24^. Using this method, two of the patients (patients 2 and 10) had decreased steatosis (4.49- and 6.20-fold, respectively) and three patients (patients 4, 7, and 8) had increased steatosis (7.34-, 2.22-, and 3.26-fold, respectively) post-DAA treatment (Table 2, Fig. 1). Representative examples that showed an increase (patient 2) and a decrease (patient 4) in the percentage of hepatic steatosis after treatment, using the Visiopharm steatosis algorithms, are shown in Fig. 3. Although the steatosis assessments made by the pathologist were mostly in agreement with the Visiopharm software regarding the relative increases and decreases in each patient’s pre- and post-treatment liver biopsies (Table 2, see patients 2, 4, and 10), the imaging analysis algorithm showed much smaller percentages of hepatic steatosis overall and was better at detecting subtle variations (Table 2, see patients 7 and 8).

**Figure 4:**
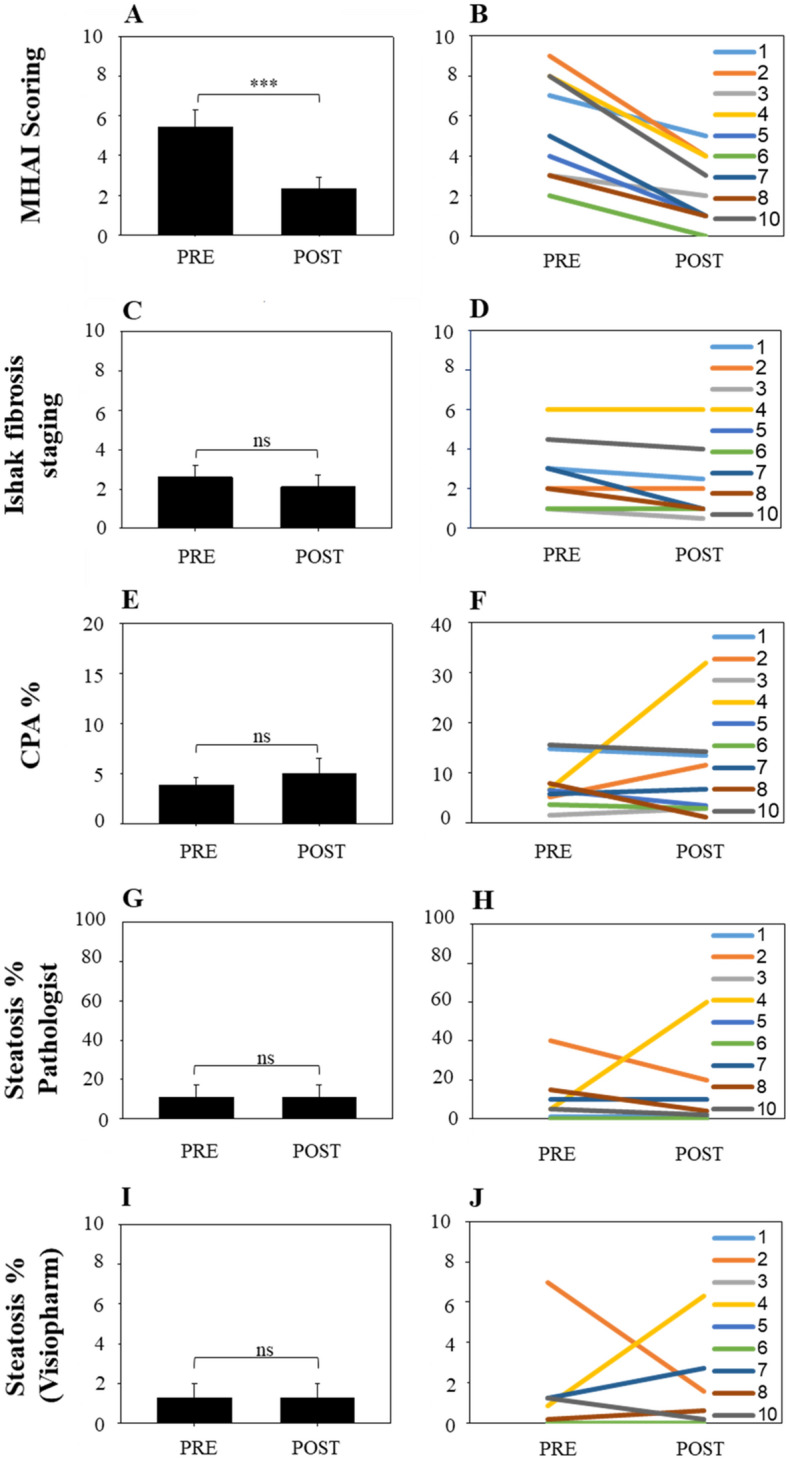
Comparison of histopathologic changes in patient liver biopsies before and after DAA treatment. Liver biopsies from the study patients (n = 10) were evaluated for inflammation, steatosis, and fibrosis by a board-certified pathologist. We also compared differences in the CPA and percent of steatosis (using Visiopharm) before and after treatment. (**A**) The MHAI was used to grade the amount of hepatitis activity in each patient’s liver biopsy^25^. Liver biopsies post-DAA treatment had significantly decreased MHAI scores (P < 0.001). (**B**) We then compared changes in each patient’s individual MHAI scores pre- and post-DAA treatment. Even though all the patients showed decreased MHAI scores, 60% of the patients still had mild, persistent lymphocytic inflammation. (**C**) Next, we compared the stages of hepatic fibrosis using the Ishak criteria (scale of 0 to 6) as determined by the pathologist. No significant difference was observed between pre- and post-DAA treatment (P < 0.063). (**D**) The changes in hepatic fibrosis are shown for each individual patient. (**E**) There were no significant differences in the CPAs between the patients before and after treatment. However, when the patients were evaluated individually (**F**), patient 4 showed a marked increase in fibrosis in her post-treatment liver biopsy when compared to her initial biopsy (yellow line). (**G**) Next, we compared differences in the amount of steatosis in the patients’ pre- and post-DAA liver biopsies using a custom algorithm from Visiopharm, which calculated the amount of steatosis per total biopsy area (see Fig. 2). The majority of patients had decreased or similar amounts of steatosis in their post-treatment liver biopsy when compared to the one collected prior to treatment. However, since Patient 4 had such a marked increase in hepatic steatosis in her post-treatment biopsy, no significant difference was observed. This was best highlighted when the patients were compared on an individual basis (**H**). *Significant differences between values pre- and post- DAA treatment (P value < 0.05). The colored lines include patients 1 through 8 and 10. ns: not significant. MHAI: Modified hepatitis activity index; Pre: pre-DAA treatment; Post: post-DAA treatment; CPA: Collagen proportionate area, “Eq. (1)”.

Next, we compared all the patients’ MHAI scores, Ishak fibrosis stages, CPA, and hepatic steatosis percentages before and after treatment with DAAs (Fig. 4). Figure 4 also shows the changes for each of these parameters in each individual patient’s pre- and post-treatment liver biopsy. The MHAI scores significantly decreased after treatment, with a mean of 5.4 and 2.3, in the pre- and post-treatment biopsies, respectively (*P* < 0.001) (Fig. 4A, Table 2). Comparison of Ishak fibrosis stages pre- and post-treatment did not show significant differences, with a mean of 2.6 and 2.1, respectively (P = 0.063) (Fig. 4C). Although the combined CPA calculations were not significantly different between the pre- and post-treatment groups (P = 1.0) (Fig. 4E), they showed more striking differences when the biopsies were compared on an individual basis for each patient before and after treatment (Fig. 4F). The majority of patients had decreased steatosis in their post-treatment liver biopsies when compared to the one collected prior to treatment (Fig. 4G–J). However, since patient 4 had such a marked increase in hepatic steatosis in her post-treatment biopsy, no significant difference was observed (P > 0.05).

### Clinical outcome

All patients achieved SVR in the study, regardless of regimen, and transaminases returned to baseline (Tables 1 and 3). Serum ALT levels decreased from a mean of 105.1 U/L (median, 65 U/L; SD, ± 108.7) to a mean of 29 U/L (median, 28.5 U/L; SD, ± 10.3; *P* = 0.015). Aspartate aminotransferase (AST) decreased from a mean of 96.5 U/L (median, 59.5 U/L; SD, ± 104.6) to a mean of 27 U/L (median, 25 U/L; SD, ± 7.4; *P* = 0.013). Alkaline phosphatase (ALP) levels decreased from a mean of 117.3 U/L (median, 115.5 U/L; SD, ± 34) to a mean of 104.2 U/L (median, 111 U/L; SD, ± 26.3; *P* = 0.033). Two patients (patients 2 and 10) had persistently elevated ALP after DAA treatment (Table 3). Two patients (patients 2 and 8) were co-infected with HIV (> 75 copies/ml) at the time of pre-DAA treatment. However, after clinical follow-up (1–3 years) these patients had undetectable HIV copies (Table 4).

**Table 3:** Evaluation of liver injury and function pre- and post-DAA therapy.

HCV pt #	Blood^a^	ALT (9–51 U/L)	AST (13–40 U/L)	ALP (34–122 U/L)	Albumin (3.5–5.0 g/dL)	Total bili (0.1–1.1 mg/dL)	Platelets (150–328 × 10^3^/μL)	PT (12.0–14.7 s)	INR (< 1.1)
1	Pre-Tx	90**	66**	93**	4.6	0.3	392**	14.3	1.1
	Post-Tx	27	22	77	4.0	0.3	358**	12.4	0.9
2	Pre-Tx	352**	361**	140**	4.1	0.8	157	13.4	1.0
	Post-Tx	22	16	136**	4.0	0.5	223	12.6	0.9
3	Pre-Tx	82**	70**	128**	4.2	0.3	276	12.7	1.0
	Post-Tx	39	29	108	4.7	0.7	259	12.9	1.0
4	Pre-Tx	239**	168**	94	4.3	1.0	101**	15.4**	1.2**
	Post-Tx	33	38	78	4.0	0.9	121**	14.4	1.1
5	Pre-Tx	40	29	108	4.4	0.5	322	12.7	0.9
	Post-Tx	25	25	114	4.9	0.1	344**	12.7	1.0
6	Pre-Tx	28	30	123**	4.1	0.7	197	13.3	1.0
	Post-Tx	23	33	114	4.3	0.5	198	13.7	1.1
7	Pre-Tx	48	53**	138**	4.4	0.8	194	12.2	0.9
	Post-Tx	30	25	120	4.0	0.3	210	13.8	1.1
8	Pre-Tx	15	23	99	4.0	0.5	159	12.6	0.9
	Post-Tx	33	24	105	4.5	0.6	200	12.3	0.9
9	Pre-Tx	35	32	63	4.4	0.6	145**	13.0	1.0
	Post-Tx	48	38	55	4.3	0.9	240	12.7	1.0
10	Pre-Tx	122**	133**	187**	4.3	0.8	179	13.2	1.0
	Post-Tx	10	20	135**	4.0	0.5	247	13.8	1.1
*P value		0.015	0.013	0.033	0.940	0.316	0.057	0.634	0.798

DAA: direct-acting antiviral; HCV: hepatitis C virus; Pt: patient; Pre-Tx: pre-DAA treatment; Post-Tx: post-DAA treatment; ALT: alanine aminotransferase; AST: aspartate aminotransferase; ALP: alkaline phosphatase; Bili: Billirubin; PT: prothrombin time; INR: international normalized ratio; U/L: Units per liter; g/dL: grams/deciliter; mg/dL: milligrams/deciliter.

*P values < 0.05 were considered statistically significant.

**Abnormal values.

^a^Blood was collected from patients with HCV pre- and post-DAA treatment.

**Table 4:** Clinical outcome of study patients.

Pt #	Data collection	HIV viral load (copies/mL)	Cancer	Ascites	SBP	Varices	Splenomegaly	Jaundice	Icterus	Encephalopathy		Coagulopathy	HRS	HPS
1	Pre-Tx	nd	No	No	No	No	No	No	No		No	No	No	No
	Post-Tx	nd	No	No	No	No	No	No	No		No	No	No	No
	1-3 dfu	Not detected	No	No	No	No	No	No	No		No	No	No	No
2	Pre-Tx	2471.0	No	No	No	No	Yes^c^	No	No		No	No	No	No
	Post-Tx	Not detected	No	No	No	No	nd	No	No		No	No	No	No
	1-3 dfu	nd	nd	nd	nd	nd	Yes^c^	nd	nd		nd	nd	nd	nd
3	Pre-Tx	< 75	No	No	No	No	No	No	No		No	No	No	No
	Post-Tx	Not detected	No	No	No	No	No	No	No		No	No	No	No
	1-3 dfu	Not detected	No	No	No	No	No	No	No		No	No	No	No
4^a^	Pre-Tx	nd	nd	nd	nd	nd	nd	nd	nd		nd	Yes^c^	nd	nd
	Post-Tx	nd	LI-RADs 2	No	No	Yes^c^	Yes^c^	No	No		Yes^c^	No	No	No
	1-3 dfu	Not detected	HCC^b^	Yes^c^	No	Yes^c^	Yes^c^	No	No		Yes^c^	No	No	No
5	Pre-Tx	Not detected	No	No	No	No	No	No	No		No	No	No	No
	Post-Tx	Not detected	No	No	No	No	No	No	No		Yes^c^	No	No	No
	1-3 dfu	Not detected	No	No	No	No	No	No	No		Yes^c^	No	No	No
6	Pre-Tx	< 75	No	No	No	No	No	No	No		No	No	No	No
	Post-Tx	Not detected	No	No	No	No	No	No	No		No	No	No	No
	1-3 dfu	Not detected	No	No	No	No	No	No	No		No	No	No	No
7	Pre-Tx	< 75	No	No	No	No	Yes^c^	No	No		No	No	No	No
	Post-Tx	Not detected	No	No	No	No	Yes^c^	No	No		No	No	No	No
	1-3 dfu	Not detected	No	No	No	No	Yes^c^	No	No		No	No	No	No
8	Pre-Tx	27,008	No	No	No	No	No	No	No		No	No	No	No
	Post-Tx	Not detected	No	No	No	No	No	No	No		No	No	No	No
	1-3 dfu	Not detected	No	No	No	No	No	No	No		No	No	No	No
9	Pre-Tx	< 40	No	No	No	No	No	No	No		No	No	No	No
	Post-Tx	66	No	No	No	No	No	No	No		No	No	No	No
	1-3 dfu	Not detected	No	No	No	No	Yes^c^	No	No		No	No	No	No
10^a^	Pre-Tx	Not detected	No	No	No	No	No	No	No		No	No	No	No
	Post-Tx	Not detected	No	No	No	No	No	No	No		No	No	No	No
	1-3 dfu	Not detected	Cholangiocarcinoma^b^	No	No	No	No	No	No		Yes^c^	No	No	No

Pt: patient; Pre-Tx: pre-DAA treatment; Post-Tx: post-DAA treatment; dfu: duration of follow up; LI-RADs: liver imaging reporting and data system; SBP: spontaneous bacterial peritonitis; HRS: hepatorenal syndrome; HPS: hepatopulmonary syndrome; nd: not done.

^a^Deceased patients.

^b^Patients that developed liver cancer post-DAA treatment.

^c^Abnormal clinical outcomes.

To assess for markers of liver injury and function, we documented levels of hepatic enzymes, albumin, prothrombin time (PT)/international normalized ratio (INR), and total bilirubin (Table 3). To determine if portal hypertension was likely, we documented platelet numbers and the presence or absence of ascites, esophageal varices, splenomegaly, and other manifestations of end-stage liver disease (Tables 3 and 4). Patient 4 had mild thrombocytopenia and an elevated PT (15.4 s) that improved after DAA treatment. Even though most of the patients had platelets in the normal range prior to treatment, the levels slightly increased after treatment, with means of (212.2 and 240 × 10^3^/µL), respectively (*P* = 0.057). Patient 1 also had mild, persistent thrombocytosis pre- (392 × 10^3^/µL) and post- (358 × 10^3^/µL) DAA treatment (Table 3). Despite achieving SVR, several patients had persistent or new development of splenomegaly (patients 2, 4, 7 and 9). We also evaluated the patients for evidence of hepatic decompensation and other complications of chronic liver disease, including the presence or absence of ascites, spontaneous bacterial peritonitis, esophageal varices, jaundice, scleral icterus, hepatic encephalopathy, hepatorenal syndrome, and hepatopulmonary syndrome (Table 4). Three patients developed hepatic encephalopathy at follow-up (patients 4, 5, and 10). All patients had normal albumin and total bilirubin levels pre- and post-treatment.

Patient 4, a non-Hispanic Caucasian female that had an increased BMI, was the only patient who had well-developed cirrhosis at the time of her first biopsy. This patient started alpha-interferon/Ribavirin approximately three years after her first biopsy was obtained and failed treatment. Two years later, she was approved for DAA treatment and was given Sofosbuvir/Ribavirin for a duration of 12 weeks and successfully achieved SVR. Even though her liver enzymes initially normalized (Table 3), her post-treatment liver biopsy showed a 4.7-fold higher percentage of fibrosis using the CPA calculation and a 7.34-fold increase in macrovesicular steatosis (Table 2 and Figs. 1, 2, 3, 4). Her post-treatment liver biopsy also showed large areas of parenchymal extinction that had substantially increased from her initial, pre-treatment biopsy (Figs. 1 and 2). Two years after achieving SVR, she developed several clinical manifestations of portal hypertension and decompensated liver disease, including ascites, esophageal varices, splenomegaly, and hepatic encephalopathy (Table 4). Initial screening for HCC by CT scan post-treatment showed a liver lesion that was “probably benign” [i.e., a Liver imaging reporting and data system (LI-RADS) score of 2], however, HCC (LI-RADS score of 5) was diagnosed during clinical follow up at 3.5 years post-treatment and the patient died approximately 6 years after achieving SVR. A single patient (patient 10) developed cholangiocarcinoma with metastasis, two years after finishing her DAA treatment regimen, and died 4 years after achieving SVR.

## Discussion

All study patients achieved SVR and had undetectable loads post-treatment (Table 1). Even though 60% of patients had persistent portal lymphocytic inflammation post-treatment, there was still significantly decreased hepatic inflammation as determined by the MHAI scores (P = 0.001) (Table 2, Figs. 1 and 4). The study patients did not have significant differences in the amount of fibrosis pre- and post-treatment regardless of whether this was determined by a pathologist using the Ishak fibrosis staging system^25^ or by the calculated CPA^19,26^ (Table 2). Hepatic macrovesicular steatosis was the most variable histopathologic finding pre-and post-DAA treatment, with some patients showing decreases and others showing increases (Table 2, Figs. 3 and 4). Regardless of the histopathologic findings in the patients’ post-treatment liver biopsies, all the patients had transaminases and alkaline phosphatase levels in the normal range after achieving SVR.

While hepatic steatosis can be caused by HCV infection and should theoretically resolve after treatment, its persistence is an important factor that may enhance liver injury and progression of fibrosis^27^. This is especially true in patients with other risk factors for development of hepatic steatosis/steatohepatitis, such as metabolic syndrome or diabetes^28^. Some studies have shown that patients that have persistent steatosis after treatment have a high prevalence of developing cirrhosis^6^ and HCC^29^. In patients with non-genotype 3, like the patients in this study, steatosis is more often correlated with BMI and visceral fat distribution^27^. The patients in this study that had the most hepatic steatosis in their post-treatment liver biopsies all had BMIs greater than 30 (Table 1; patients 2, 4, and 7). The presence of steatosis in some of the study patients could have also contributed to the persistent inflammation^30^. In addition to the amount of steatosis in liver biopsies, other parameters associated with the progression of fibrosis include HCV genotype 3 infection itself, increased necro-inflammatory activity (e.g., increased MHAI scores), and elevated serum alanine aminotransferase (ALT)^31^.

An interesting observation was the marked difference in the percentage of hepatic steatosis in the estimation made by the pathologist when compared to that determined using the Visiopharm algorithm. Visiopharm is an advanced imaging software platform that enables more precise quantification of various parameters such as fibrosis and steatosis in histology material^22,24,32^. Overall, the percentages estimated by the pathologist were much higher than those determined by the imaging software. One explanation for this difference is that the pathologist estimates the percentage of hepatocytes that appear to be involved by lipid droplets by visualization through the microscope. The Visiopharm algorithm uses the percent of lipid droplets (i.e. round holes in the tissue) divided by the entire tissue area. For the most part, the trends were similar and both methods were able to detect increases or decreases in hepatic steatosis after treatment. Imaging modalities, such as Visiopharm, offer a much more objective, and likely more accurate, assessment of certain histopathologic features. Calculation of the CPA was also useful and was able to show differences in the severity of cirrhosis. Even though Patient 4’s pre- and post-treatment biopsies both reported Ishak fibrosis stages of 6 out of 6, the CPA showed a substantial increase in the amount of collagen in the follow-up biopsy (32.0%) when compared to the first (6.7%) (Table 4).

A major strength of this study was that it evaluated histopathologic features in 10 post-treatment liver biopsies from patients that achieved SVR after treatment with DAAs. While several clinical studies have been conducted, very few have evaluated histopathologic changes in post-treatment liver tissue. A few other studies have looked at the differences in inflammation, immune activation, and fibrosis after DAA therapy. These included evaluations of HCV patients^30^, HCV/HIV co-infected patients^33^, HCV/AIH patients^34^, HCV patients prior to liver transplantation^35^, and allograft liver biopsies from patients transplanted for chronic hepatitis C^13^. In the present study, persistent inflammation (MHAI scores > 2) was observed in 60% of the patients after treatment (Table 2, Figs. 1 and 4). It is thought that improvement in hepatic inflammation becomes more evident when the post-treatment liver biopsy is collected several years after completing DAA therapy. Interestingly, the patient with the longest duration between achieving SVR and the collection of her post-treatment biopsy (28.4 months), still had persistent inflammation. Of note, she was a patient who had previously failed treatment with alpha interferon/ribavirin prior to achieving SVR with sofosbuvir/ribavirin, which may have been a contributing factor. Persistent lymphocytic inflammation has been reported to last up to 16.5 years after HCV cure in response to alpha-IFN/ribavirin antiviral therapy^33,35^. We do not yet have long-term follow-up data from patients that were treated exclusively with IFN-free regimens, like the rest of the patients in this study.

Viral persistence in the liver has also been suggested as a cause of ongoing hepatic inflammation^36^. However, in the absence of virus, ongoing inflammation is proposed to be immunologically driven^35^. Some studies have reported that this inflammation has no effect on patient outcomes, while others propose that it causes fibrosis persistence^37^ and the development of HCC^38^. Observations that helped distinguish the persistent inflammation in this study from other types of active hepatitis included that the inflammation was isolated to the portal tracts, the biopsies lacked interface and lobular activity, and that all the patients had normal transaminases. It is important for clinicians and pathologists to be aware that a large number of patients will have persistent inflammation confined to the portal tracts after DAA treatment and this does not always indicate that another type of hepatitis is present.

In Western countries, HCV is the most common cause of HCC^39^. A few smaller studies have shown that DAA therapy does not appear to reduce the risk of HCC as much as IFN-based regimens^9,40^. However, more recent larger studies have shown that HCV-infected and HCV/HIV co-infected patients that had cirrhosis and achieved SVR post-DAA treatment had up to a 71% reduction in HCC risk^41–43^. With both IFN and IFN-free therapies, if SVR is achieved, decreased hepatic inflammation and activity scores have been reported^44^. IFN-based studies have shown that patients with persistent inflammation and without fibrosis regression are still at high risk for HCC after SVR^38,45,46^. DAA therapies have only been available since 2011, therefore, long term studies of HCC development in the presence of persistent inflammation and fibrosis are limited when compared to those with IFN-based regimens. Short term studies seem to suggest that the presence of increased inflammation and fibrosis are associated with hepatocarcinogenesis^37^. Discrepancies in the literature may be explained by differences in patient demographics, such as older age, presence of other risk factors such as diabetes mellitus, and those who had impaired liver function with less frequent screening^47^. Regardless, the evidence is clear that patients with advanced fibrosis or cirrhosis have a persistent risk of HCC development, although this risk is lower in patients who achieve SVR. Patients with cirrhosis that are male or have high liver stiffness, increased alpha-feto protein levels, or coexisting diabetes, seem to be the most at risk^18^. Despite this known risk, the most recent AASLD guidelines do not specifically discuss surveillance recommendations for patients with cirrhosis that achieved SVR after DAAs (AASLD practice guidelines/http://www.hcvguidelines.org). However, they do recommend that all patients with cirrhosis should undergo surveillance^39^.

Occasionally, patients with chronic hepatitis C can also have laboratory and histologic features that suggest superimposed autoimmune hepatitis. Recent reports have shown that most of these patients respond to DAA therapy and have normal transaminases after treatment^34,48^. Thus, the serologic and/or histopathologic features of autoimmune hepatitis that are observed in patients with chronic hepatitis C are most likely due to the virus rather than a primary immune process. Regardless, fibrosis stage is the most important histologic factor to predict mortality and liver-related complications^49^. Studies have shown mixed results regarding the amount of hepatic fibrosis, with some reporting worsening of fibrosis after treatment (from stages 3 to 4)^48^, while others have shown regression (from stages 4 to 3)^34,48^. A longer duration prior to collection of the post-treatment biopsy may be needed to see evidence of regression^6^.

We realize that a limitation of this study is the decreasing frequency in which liver biopsies are used to determine the stage of fibrosis in patients with HCV. Non-invasive techniques, such as elastography, are increasingly used to assess hepatic steatosis and fibrosis^50–54^. Even though some of these do not perform as well as liver biopsy for detection of early or intermediate stages of fibrosis, they are quite accurate for diagnosing advanced fibrosis (bridging fibrosis and cirrhosis) in patients with chronic hepatitis C^52–54^. When liver biopsies are unavailable, non-invasive methods should be used to determine if a patient has advanced fibrosis or other risk factors for fibrosis progression such as hepatic steatosis, since this study further supports their presence as risk factors for poor clinical outcome^6,9^. In this study, however, Fibroscan would not have been able to detect the presence of persistent inflammation, changes in hepatic steatosis, CPA, or the intermediate stages of fibrosis identified in several patients (Table 2).

One of the most important observations made in this study was the development of primary liver cancer in two of the patients (patients 4 and 10), despite achieving SVR (Table 4). These two patients were diagnosed with HCC and cholangiocarcinoma 46 and 35 months after achieving SVR, respectively. Importantly, these patients were those with the most advanced fibrosis in their baseline biopsies; patient 4 already had cirrhosis (fibrosis stage: 6/6) and patient 10 had well-developed bridging fibrosis (stage 4–5/6) (Table 2). Patient 4 had multiple risk factors for the development of HCC^55,56^. She was obese with hyperlipidemia and uncontrolled diabetes mellitus, had failed previous treatment with alpha-IFN/ribavirin prior to successful treatment with DAAs, developed increased macrovesicular steatosis with active steatohepatitis in her follow-up biopsy, and showed markedly increased hepatic fibrosis by the calculated CPA (Table 2). AASLD guidelines recommend that all patients with cirrhosis should undergo surveillance for cancer development, especially HCC^39^. Patient 10 had well-developed bridging fibrosis at the time of her baseline biopsy and later developed small duct, intrahepatic cholangiocarcinoma. HCV infection itself is a known risk factor for this type of liver cancer^57–59^. Reports have suggested that patients with cirrhosis should be evaluated every 6 months by ultrasound with or without alpha fetoprotein measurements, as recommended in the recent AASLD guidelines^60,61^.

One of the main strengths of this study was the ability to compare both pre- and post-treatment liver biopsies from the same patient. Liver biopsies are able to accurately stage disease and remain the gold standard for assessment of hepatic fibrosis^17^. Obtaining liver tissue also allows for more objective quantitative measurements using imaging software platforms such as Visiopharm^22,24,32^ and evaluation of the hepatic microenvironment using newer techniques such as spectral imaging^26,62^. The main limitation of this study was the small number of patients. This was due to the lack of availability of post-treatment biopsies. Since histologic evaluation of liver biopsies after DAA therapy is no longer considered standard of care, these specimens will become even more infrequent. However, when liver biopsies are collected in clinical practice, pathologists should provide thorough reports that implement newer imaging analysis approaches (e.g., Visiopharm), whenever possible. The short duration between DAA treatment completion and collection of their follow-up liver biopsies, which ranged between 6.0 and 28.4 months (Table 2), was another limitation of the study and may explain why we did not detect regression of fibrosis.

## Conclusion

The results of this study suggest that all patients with bridging fibrosis (i.e., F3 fibrosis and beyond) should be screened regularly for the development of primary liver cancers, including HCC and cholangiocarcinoma. It would also be beneficial to encourage patients with advanced fibrosis to avoid other causes of chronic liver injury, such as alcohol use and risk factors for developing superimposed hepatic steatosis/steatohepatitis. Clinicians and pathologists should be aware that persistent inflammation is quite common. Thus, an unnecessary work-up for other causes of chronic active hepatitis should avoided, particularly when the lymphocytic infiltrate is isolated to the portal tracts in patients with normal transaminases. Despite all 10 patients in this study achieving SVR and having normal levels of hepatic enzymes at follow-up, biopsies from some patients showed increased steatosis and fibrosis at follow-up, and two patients developed cancer. In summary, patients that have advanced fibrosis and other risk factors for chronic liver disease, such as obesity and diabetes, should be considered high risk, and thus, should still be followed closely and screened for primary liver cancers and other manifestations.

## Materials and methods

### Study patients and DAA treatment

The University of Texas Medical Branch (UTMB), Institutional Review Board approved this retrospective clinical study, where we retrospectively evaluated liver biopsy tissues that were collected from HCV-infected patients before (pre-) and after (post-) DAA treatment. All patients had to have achieved SVR after DAA therapy for chronic HCV infection (see Table 1). Although we had several patients that had pre-treatment biopsies available, only 10 had post-treatment biopsies collected with adequate clinical follow-up. We included all ages, women, and minorities in the study. Although children were not excluded, at the time of this submission no children under the age of 18 met the inclusion criteria. Liver biopsies were collected from January 2008 to June 2017 and histopathological observations were correlated with clinical outcomes by review of the medical record.

At the time of this publication, 10 HCV+ patients, from a total cohort of 101, had pre- and post-treatment biopsies available. Demographic data, HCV viral status, DAA regimen and treatment duration were recorded for each patient (see Table 1). The patients were treated according to international and national guidelines^63^ and several different DAA regimens/combinations were administered for up to 12 weeks. Patients with HCV genotype 1a or 1b were treated with either sofosbuvir/simeprevir, sofosbuvir/ledipasvir or Viekira Pak. HCV genotypes 2 or 2b were treated with sofosbuvir/ribavirin or sofosbuvir/simeprevir/ribavirin (see Table 1)^64^. All of the HCV/HIV coinfected patients in this study were on highly active anti-retroviral therapy (HAART) and only patients 2 and 8 had detectable HIV viral loads at the time when their baseline biopsies were collected. All the patients had undetectable HIV viral loads when their follow-up biopsies were obtained. None of the patients had serologic evidence of coinfection with other hepatotropic viruses (e.g., hepatitis A, B or E) or elevation of autoimmune-related serologic markers during the duration of the study.

All patients had paired pre- and post-treatment liver biopsies, except for patient 9, who did not have a pre-treatment liver biopsy collected. Patients were retrospectively followed after DAA treatment to evaluate for SVR and normalization of liver enzymes. Biopsies were evaluated for persistence of inflammatory activity, residual steatosis and stage of fibrosis. SVR was defined as undetectable plasma HCV RNA after 12 weeks post-DAA treatment completion. Written informed consent was obtained from all study patients and the data collected was de‐identified.

### Liver biopsy collection and whole slide imaging

Each patient had two percutaneous core needle biopsies obtained, one prior to DAA treatment and one at follow up (ideally within 1–2 years post-treatment). Liver biopsies were obtained as standard of care by licensed radiologists via the percutaneous route using an 18-gauge core needle. After collection, the tissue was immediately placed into 10% buffered formalin, processed using a TissueTekVIP tissue processor, paraffin-embedded, and sectioned at 3 µm using a Thermo-Fisher CryoStar NX70 cryostat. Hematoxylin an eosin (H&E) and Masson’s trichrome stains were conducted on a Ventana Ultra automated stainer (Roche Diagnostics, Tucson, Arizona). All processing was performed in a College of American Pathologists (CAP)-accredited laboratory by licensed histotechnologists. Each patient’s pre- and post-DAA treatment liver biopsies (n = 10) were evaluated by a board-certified liver pathologist (H.S.L) who was blinded to the patient’s serology, clinical history, and prior pathology reports. Biopsies were evaluated using established numerical scoring systems for histologic features of hepatitis inflammatory activity staging of fibrosis and grading of steatosis^25,65,66^. The modified hepatitis index (MHAI) evaluates several parameters of hepatic inflammatory activity, including periportal/periseptal interface hepatitis, confluent necrosis, focal lytic necrosis, apoptosis/inflammatory activity, and portal inflammation^25^. The stage of fibrosis was first determined using the Ishak criteria^25^. Whole biopsy images were obtained for each patient’s liver biopsy using an Aperio Image Scope Digital slide scanner (Leica Biosystems, Buffalo Grove, IL).

### Quantification of the collagen proportionate area (CPA)

We calculated the CPA as previously published^19,26^. Briefly, Masson’s trichrome stains were digitally scanned on an Aperio ImageScope Digital slide scanner (Leica Biosystems, Wetzlar, Germany) and low-power (×1.2) images of the entire surface area of each core were measured in number of pixels using Nikon’s NIS Elements-BR Imaging Software (Tokyo, Japan). Next, blue-stained areas from the trichrome stain were identified and thresholds were set using the software. The fibrotic area was divided by the total biopsy surface area to obtain the percent fibrosis

1 $$ \left( {\% {\text{ fibrosis}} = \left[ {\Sigma {\text{fibrotic area in pixels}}/\Sigma {\text{ total area in pixels}}} \right]{\text{ }} \times {\text{ 1}}00} \right). $$

Liver capsules and large portal tracts (identified by presence of nerve bundles) were excluded.

### Quantification of hepatic steatosis

We used a modified custom-trained deep learning algorithm from Visiopharm (Visiopharm Corp, version 2019.04; Westminster CO, US) to identify steatosis in patient liver biopsies. This imaging software platform has been used to more objectively measure steatosis and fibrosis in several other studies^22–24^. H&E stains were digitally scanned on an Aperio ImageScope Digital slide scanner at low-power (1.2×) and images that included the entire surface area of each biopsy were analyzed. The algorithm was trained to distinguish hepatic steatosis from other common features of liver tissue, such as blood vessels and tissue tears from tissue processing. The image classes generated by the deep learning network were then combined with another feature of the software that separates fat droplets using their round shape. All fat droplets larger than 250 square microns were considered to be macrovesicular steatosis, while those smaller were considered to be microvesicular steatosis. The large (i.e., macrovesicular) and small (i.e., microvesicular) droplet areas were then combined to give the total area of steatosis, which was divided by the total biopsy tissue area using the automatic tissue detection feature in Visiopharm software. In summary, the percent fat for each patient’s liver biopsy was calculated by dividing the total fat area by the total area of liver biopsy and then multiplying that result by 100. i.e.,

2 $$ \% {\text{ steatosis }} = {\text{ }}\left( {\sum {\text{steatosis area in pixels}}/\sum {\text{ total area in pixels}}} \right){\text{ }} \times {\text{1}}00. $$

All results were exported to Excel (Microsoft, Inc.) for further analysis.

### Laboratory tests

We compared laboratory studies (ALT, AST, ALP, albumin, bilirubin, platelets prothrombin time, and INR) in pre- and post-treatment patients (n = 10). HCV viral load/genotype and liver enzymes were determined in blood following standard molecular procedures in-house, in a CAP and Clinical Laboratory Improvement Amendments (CLIA)-accredited clinical chemistry laboratory at UTMB. An FDA-approved protocol for the HCV quantitation in plasma/serum samples of infected individuals was done using an Abbott m2000 Real Time reverse transcription-polymerase chain reaction (RT-PCR) assay. The FDA-approved dynamic range for the HCV test is 12 IU/ml to 100,000,000 IU/ml (1.08–8.00 Log IU/ml). The HIV tests were conducted using two different methods. Some patients were tested using an HIV branched-chain DNA quantitative assay (Bayer Corporation, Pittsburgh, PA). This test had a dynamic range of 75–500,000 copies/mL A positive result was a value greater than or equal to 400 copies/mL. An Abbott m2000 RT-PCR assay was used for most patients. This test had a dynamic range of 40 to 10,000,000 copies/ml (1.60–7.00 Log copies/ml). A positive result was a value greater than 40 copies/mL. Assay results were reported in copies/ml; 1 copy = 1.74 IU (International Units). Genotypes were determined using a RT-PCR, line probe assay (LiPA) on patients with confirmed HCV disease. Laboratory data were collected for each patient at the time of HCV diagnosis and between 3 and 4 months post-DAA treatment.

### Statistical analysis

Data were analyzed using SigmaPlot software (Systat Software Inc., San Jose, CA). Descriptive statistics (mean, median, and SD) were obtained and P values were determined using the Student’s two-tailed t test (after Shapiro–Wilk normality). When indicated, the non-parametric Wilcoxon rank sum test (also called Wilcoxon–Mann–Whitney test) was used to compare the steatosis grading and fibrosis staging between the two patient groups (control, pre- and post-DAA treatment). P < 0.05 was chosen for significance.

## Data Availability

Data analyzed during the current study is included in the tables.
